# Prognostic Nomograms for Predicting Overall Survival and Cancer-Specific Survival of Patients With Early Onset Colon Adenocarcinoma

**DOI:** 10.3389/fonc.2020.595354

**Published:** 2020-10-20

**Authors:** Huimin Jin, Yuqian Feng, Kaibo Guo, Shanming Ruan

**Affiliations:** ^1^First Clinical Medical College of Zhejiang Chinese Medical University, Hangzhou, China; ^2^Department of Medical Oncology, The First Affiliated Hospital of Zhejiang Chinese Medical University, Hangzhou, China

**Keywords:** nomogram, overall survival, cancer-specific survival, early onset colon adenocarcinoma, prognosis, Surveillance; Epidemiology and End Results database

## Abstract

**Background:**

The incidence of colon cancer in young patients is on the rise, of which adenocarcinoma is the most common pathological type. However, a reliable nomogram for early onset colon adenocarcinoma (EOCA) to predict prognosis is currently lacking. This study aims to develop nomograms for predicting the overall survival (OS) and cancer-specific survival (CSS) of patients with EOCA.

**Methods:**

Patients diagnosed with EOCA from 2010 to 2015 were included and randomly assigned to training set and validation set. Cox regression models were used to evaluate prognosis and identify independent predictive factors, which were then utilized to establish the nomograms for predicting 3- and 5-year OS and CSS. The discrimination and calibration of nomograms were validated using the calibration plots, concordance index, receiver operating characteristics curve, and the decision curve analysis.

**Results:**

A total of 2,348 patients were screened out, with 1,644 categorized into the training set and 704 into the validation set. Multivariate analysis demonstrated that gender, age, tumor size, T stage, M stage, regional node, tumor deposits, lung metastasis and perineural invasion were significantly correlated with OS and CSS. The calibration plots indicated that there was good consistency between the nomogram prediction and actual observation. The C-indices for training set of OS and CSS prediction nomograms were 0.735 (95% CI: 0.708–0.762) and 0.765 (95% CI: 0.739–0.791), respectively, whereas those for validation set were 0.736 (95% CI: 0.696–0.776) and 0.76 (95% CI: 0.722–0.798), respectively. The results of ROC analysis revealed the nomograms showed a good discriminate power. The 3- and 5-year DCA curves displayed superiority over TNM staging system with higher net benefit gains.

**Conclusions:**

The nomograms established could effectively predict 3- and 5-year OS and CSS in EOCA patients, which assisted clinicians to evaluate prognosis more accurately and optimize treatment strategies.

## Introduction

Colon carcinoma is the most common malignant tumor of the digestive tract, ranking fourth in deaths from malignant tumors worldwide. In the United States, it is estimated that approximately 104,610 colon cancer cases will be diagnosed in 2020, which corresponds to 287 new cases diagnosed per day on average (1). Among all histological subtypes, colon adenocarcinoma (CA) is deemed as the most common one, accounting for 60%–70% of all cases with a poor prognosis. Although, the diagnostic methods and therapeutic approaches for the management of CA have been greatly improved in recent years, the 5-year overall survival rate remains low. Meanwhile, tumor recurrence is also one of the most daunting challenges in the clinical treatment for CA (2). A previous study reported about 70% of CA patients exhibited postoperative recurrence within 24 months after curative surgery (3). In addition, evidence from several studies showed that CA incidence varied with age. Cancer facts and figures (2020) estimated that the incidence of CA had been increasing in young adults while the overall incidence declined by 3.6% per year for older adults (≥55 years) over the last 25 years. According to the data from US National Cancer Database, the incidence increased by 2.7% annually among adults younger than age 50 in the past decade, with 75% of cases occurring aged 40 to 49. Early onset colon adenocarcinoma (EOCA) is defined as CA patients under the age of 50 at diagnosis (4). Research suggests that EOCA may share biological characteristics including poorly differentiated, highly malignant, more aggressive, mutations in mismatch repair (MMR) genes as well as high microsatellite instability (MSI-H), resulting in unfavorable prognosis (5). Concerns have been raised over the increasing incidence and the poor clinical outcomes, and it is essential to precisely identify the prognostic factors associated with EOCA and choose personalized treatment strategies.

Nomogram is widely used as a visualization method of complex mathematical models, which considers multiple risk factors, predicts the prognosis of diseases, and presents them in an intuitive way (6). However, few studies have focused specifically on the age-specific risk factors associated with prognosis. A well-structured and fully validated prognostic nomogram for EOCA patients is desired. Hence, based on sufficient registered cases from the Surveillance, Epidemiology and End Results (SEER) database, this study first delineates the major clinical and pathological characteristics of EOCA, and then establishes nomograms to predict 3- and 5-year overall survival (OS) and cancer-specific survival (CSS).

## Methods

### Data Retrieved From SEER

Clinicopathological characteristics and information of all EOCA patients were obtained from the Surveillance Epidemiology and End Results (SEER) database *via* reference number 12330-Nov2019. Supported by the National Cancer Institute, the SEER program comprehensively assembles information on cancer incidence, treatment, and patient survival since 1973 in multiple geographic regions across the United States. An ethics statement or approval is not necessary for the presented study since all of the data are publicly available and open-access. The identification of colon adenocarcinoma patients is based on the histologic/behavior code of ICD-O-3 (International Classification of Disease for Oncology, Third Edition), primary site code C18.0–C18.9, along with the cancer staging scheme (version 0204). The inclusion criteria of this study were: i) age ≤ 50 years old; ii) no missing TNM stage information; iii) with histologically proven adenocarcinoma of the colon; iv) a single primary tumor lesion (CC); v) no missing information on survival, tumor size, grade and other details; vi) not only diagnosed through autopsy or a death certificate; vii) surgery had been performed. All of included samples were randomly split into the training set and the validation set, according to the ratio 7:3. The follow-up period for entire cohort ranged from less than 1 month to 95 months (median 45, average 49.2 months). The median follow-up time was 45 months in training set and 45.5 months in validation set, respectively.

### Clinical Variables of EOCA

The demographic and clinical variables were extracted by the SEER∗Stat software (version 8.3.5), including gender, age, race, grade, tumor size, American Joint Committee on Cancer (AJCC) TNM stage, regional node, tumor deposits, perineural invasion, regional nodes status, tumor metastasis, and survival related information and cause of death. The primary endpoint was overall survival (OS), defined as the period between initial diagnosis and final follow-up or death from any cause. The second endpoint was cancer-specific survival (CSS), defined as the period from the EOCA diagnosis to the death attributed to cancer recurrence or metastasis. Age and tumor size were divided into 3 groups using the optimal cut-off value, established by X-tile bioinformatics software (Yale University, Version 3.6.1).

### Construction and Validation of Nomogram Model

The survival analysis was conducted with Kaplan-Meier method and log-rank test, while the Chi-square test was utilized for the comparison of categorical variables. Univariate Cox analysis was performed as a screening method to identify significant factors (P<0.2) for further multivariate testing. The nomogram was constructed to predict personalized survival probability based on the results from the multivariate analysis. Harrell’s concordance statistics (C-index) was applied to evaluate the discriminatory ability of the nomogram. Based on the above estimation, receiver operating characteristic (ROC) curves were drawn and their corresponding areas under the curve (AUC) were also calculated. To further assess model calibration, the calibration plot was undertaken for the measurement between observed and predicted probabilities, with a 45‐degree reference line. In addition, clinical usefulness of the nomogram models was determined using decision curve analysis (DCA) to quantify net benefit, and compared with the 7th version of TNM staging throughout the entire cohort. All the data analysis was carried out using R Software (Version 4.0.1, R Foundation for Statistical Computing). Statistically significant difference was set at P value < 0.05. However, the p-value level of 0.2 was regarded as filter value for univariate to multivariate analysis.

## Results

### Input Data From SEER

**Figure 1:**
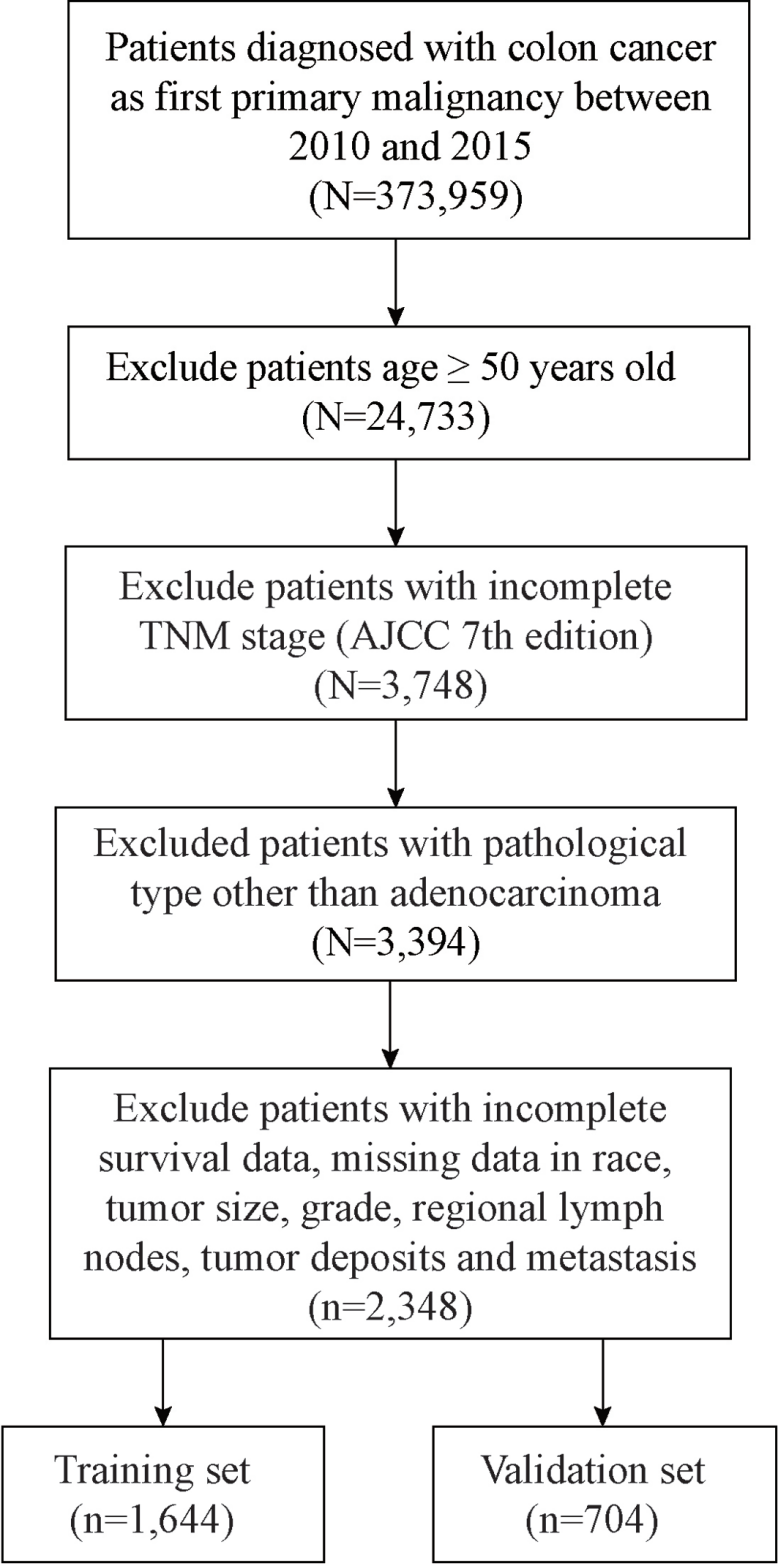
Flow diagram of the EOCA patients with training and validation sets.

In this process, a total of 2,348 patients with EOCA were screened out, of which 1,644 were assigned randomly to the training set and 704 cases were assigned to the validation cohort (**Figure 1**). Among all patients, 1,189 (50.6%) were male and 1,646 (70.1%) were the white. The most appropriate cutoff value regarding age and tumor size was selected after optimized classification by the biostatistical tool X-tile. Among the included cases, 1,425 (60.7%) were between 38–47 years old, and 1,160 (49.4%) with tumor size larger than 4.7 cm. The majority of grade is moderately (75.0%) while 83.2% were in M0 stage. The positive rate of perineural invasion was only 15.1% (negative: 84.9%) of all patients, while tumor deposits was only positive in 11.5% of all patients (negative: 88.5%). In addition, about half of the cases are regional nodes positive (52.8%). The distant metastasis occurs not often, the most common organ of metastasis is the liver (11.8%), followed by the lung (2.6%) and the bone (0.1%) (**Table 1**).

**Table 1:** Baseline demographics and clinical characteristics of the patients with early onset colon Adenocarcinoma.

Characteristics	All patients, n (%)	Training set, n (%)	Validation set, n (%)
	2,348(100.0)	1,644(70.0)	704(30.0)
Gender			
Male	1189(50.6)	821(49.9)	368(52.3)
Female	1159(49.4)	823(50.1)	336(47.7)
Age			
≤37	402(17.1)	296(12.6)	106(15.1)
38-47	1425(60.7)	988(42.1)	437(62.1)
≥48	521(22.2)	360(15.3)	161(22.9)
Race			
White	1646(70.1)	1126(68.5)	520(73.9)
Black	403(17.2)	304(18.5)	99(14.1)
Other*	299(12.7)	214(13.0)	85(12.1)
Grade			
Well	145(6.2)	104(6.3)	41(5.8)
Moderately	1760(75.0)	1245(75.7)	515(73.2)
Poorly	367(15.6)	249(15.2)	118(16.8)
Undifferentiated	76(3.2)	46(2.8)	30(4.3)
Tumor size			
≤2.4cm	296(12.6)	199(12.1)	97(13.8)
2.5-4.7cm	892(38.0)	642(39.1)	250(35.5)
≤4.7cm	1160(49.4)	803(48.8)	357(50.7)
AJCC T stage (7^th^)			
T1	218(9.28)	147(8.9)	71(10.1)
T2	265(11.3)	187(11.4)	78(11.1)
T3	1395(59.4)	981(59.7)	414(58.8)
T4a	310(13.2)	224(13.6)	86(12.2)
T4b	160(6.8)	105(6.4)	55(7.8)
AJCC N stage (7^th^)			
N0	1085(46.2)	751(45.7)	334(47.4)
N1	739(31.5)	527(32.1)	212(30.1)
N2	524(22.3)	366(22.3)	158(22.4)
AJCC M stage (7^th^)			
M0	1954(83.2)	1374(83.6)	580(82.4)
M1	394(16.8)	270(16.4)	124(17.6)
Regional nodes			
Positive	2324(98.9)	1627(99.0)	697(99.0)
Negative	24(1.1)	17(1.0)	7(1.0)
Tumor deposits			
Positive	269(11.5)	192(11.7)	77(10.9)
Negative	2079(88.5)	1452(88.3)	627(89.1)
Bone metastasis			
Yes	3(0.1)	2(0.1)	1(0.1)
No	2345(99.9)	1642(99.9)	703(99.9)
Liver metastasis			
Yes	278(11.8)	197(12.0)	81(11.5)
No	2070(88.2)	1447(88.0)	623(88.5)
Lung metastasis			
Yes	51(2.6)	40(2.4)	11(1.6)
No	2297(97.8)	1604(97.6)	693(88.5)
Perineural invasion			
Yes	354(15.1)	248(15.1)	106(15.1)
No	1994(84.9)	1396(84.9)	598(84.9)

*American Indian/AK Native, Asian/Pacific Islander. AJCC, American Joint Committee on Cancer; TNM, tumor-node-metastasis.

### Construction of Nomogram

**Figure 2:**
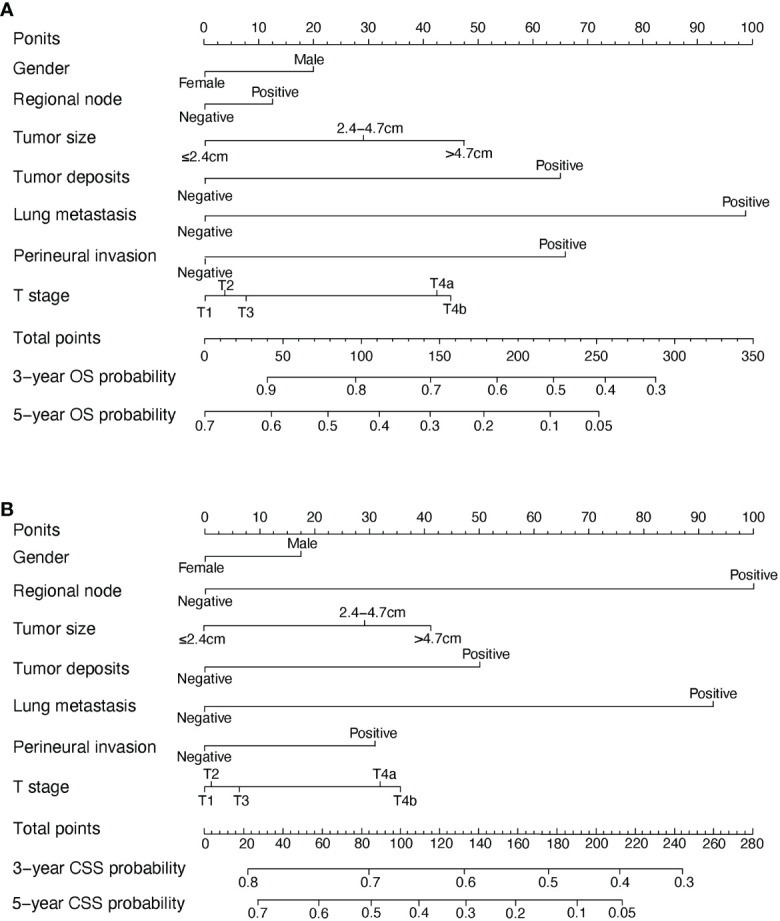
OS and CSS associated nomograms for EOCA patients. **(A)** OS nomograms for EOCA in 3- and 5-year; **(B)** CSS nomograms for EOCA in 3- and 5-year. OS, overall survival; CSS, cancer-specific survival; EOCA, early onset colon adenocarcinoma.

In the univariate COX analysis, the variables, including gender, age, tumor size, T stage, regional node, tumor deposits, lung metastasis, and perineural invasion, showed different statistic correlation with OS in EOCA patients. After adjusting for covariates, all factors listed above except age were significantly identified with OS in the multivariate COX regression (**Table 2**). The OS nomogram for predicting 3-, and 5-year overall survival rate was established by incorporating these seven independent factors (**Figure 2**). Moreover, univariate analysis demonstrated that gender, age, tumor size, T stage, M stage, regional node, tumor deposits, lung metastasis, and perineural invasion had a prominent impact on CSS in EOCA patients. These factors were subsequently included in the multivariate analysis, which showed similar results. Gender, age, tumor size, T stage, regional node, tumor deposits and lung metastasis were independently predictive of CSS and further subject to a CSS nomogram (**Table 3**, **Figure 2**).

**Table 2:** Univariate and multivariate analysis of OS in the training set (n=1,644).

Characteristics	No. of patient	Univariate analysis		Multivariate analysis	
		HR(95%CI)	*P* value	HR(95%CI)	*P* value
Gender					
Male	821	Reference		Reference	
Female	823	0.88(0.79–0.98)	0.023	0.86(0.77–0.96)	0.009
Age					
≤37	296	Reference		Reference	
38-47	988	0.89(0.77–1.03)	0.121	0.9(0.77–1.04)	0.138
≥48	360	0.91(0.77–1.09)	0.305	0.93(0.78–1.11)	0.437
Race					
White	1126	Reference		–	–
Black	304	0.95(0.82–1.11)	0.528	–	–
Other	214	0.97(0.83–1.14)	0.747	–	–
Grade					
Well	104	Reference		–	–
Moderately	1245	0.99(0.8–1.23)	0.936	–	–
Poorly	249	0.87(0.68–1.13)	0.307	–	–
Undifferentiated	46	1.09(0.72–1.66)	0.691	–	–
Tumor size					
≤2.4cm	199	Reference		Reference	
2.5-4.7cm	642	1.21(1.02–1.44)	0.03	1.39(1.11–1.73)	0.004
≤4.7cm	803	1.11(0.94–1.32)	0.217	1.28(1.02–1.62)	0.032
AJCC T stage (7^th^)					
T1	147	Reference		Reference	
T2	187	0.91(0.72–1.13)	0.386	0.77(0.59–0.99)	0.042
T3	981	0.96(0.8–1.15)	0.69	0.75(0.58–0.95)	0.02
T4a	224	0.99(0.78–1.26)	0.93	0.73(0.55–0.98)	0.039
T4b	105	1.25(0.93–1.69)	0.143	0.95(0.67–1.35)	0.792
AJCC N stage (7^th^)					
N0	751	Reference		–	–
N1	527	1(0.88–1.13)	0.992	–	–
N2	366	1.06(0.91–1.23)	0.472	–	–
AJCC M stage (7^th^)					
M0	1374	Reference		–	–
M1	270	1.12(0.91–1.38)	0.285	–	–
Regional nodes					
Positive	858	Reference		Reference	
Negative	786	0.43(0.25–0.73)	0.002	0.43(0.25–0.73)	0.002
Tumor deposits					
Positive	192	Reference		Reference	
Negative	1452	0.64(0.53–0.78)	<0.001^*^	0.67(0.55–0.82)	<0.001
Bone metastasis					
Yes	2	Reference		–	–
No	1642	59959.03(0–Inf)	0.986	–	–
Liver metastasis					
Yes	197	Reference		–	–
No	1447	0.94(0.74–1.2)	0.623	–	–
Lung metastasis					
Yes	40	Reference		Reference	
No	1604	0.42(0.24–0.75)	0.003	0.53(0.3–0.95)	0.033
Perineural invasion					
Yes	248	Reference		Reference	
No	1396	0.78(0.65–0.92)	0.004	0.82(0.68–0.98)	0.027

*Two-sided P values <0.05; HR, hazard ratio; CI, confidence intervals; AJCC, American Joint Committee on Cancer; TNM, tumor-node-metastasis.

**Table 3:** Univariate and multivariate analysis of CSS in the training set (n=1,644).

Characteristics	No. of patient	Univariate analysis		Multivariate analysis	
		HR(95%CI)	*P* value	HR(95%CI)	*P* value
Gender					
Male	821	Reference		Reference	
Female	823	0.9(0.8–1)	0.044	0.88(0.79–0.98)	0.019
Age					
≤37	296	Reference		Reference	
38–47	988	0.9(0.78–1.04)	0.156	0.9(0.78–1.04)	0.169
≥48	360	0.93(0.78–1.1)	0.407	0.95(0.8–1.12)	0.523
Race					
White	1126	Reference		–	–
Black	304	0.98(0.85–1.13)	0.762	–	–
Other*	214	0.96(0.82–1.13)	0.614	–	–
Grade					
Well	104	Reference		–	–
Moderately	1245	0.98(0.79–1.21)	0.833	–	–
Poorly	249	0.87(0.68–1.12)	0.279	–	–
Undifferentiated	46	1.07(0.71–1.62)	0.745	–	–
Tumor size					
≤2.4cm	199	Reference		Reference	
2.5–4.7cm	642	1.24(1.04–1.47)	0.014	1.4(1.13–1.74)	0.002
≤4.7cm	803	1.11(0.94–1.31)	0.213	1.28(1.02–1.6)	0.034
AJCC T stage (7^th^)					
T1	147	Reference		Reference	
T2	187	0.95(0.76–1.18)	0.625	0.8(0.62–1.02)	0.077
T3	981	0.98(0.82–1.17)	0.821	0.76(0.6–0.97)	0.028
T4a	224	1.02(0.8–1.28)	0.901	0.76(0.57–1.02)	0.064
T4b	105	1.26(0.94–1.7)	0.129	0.98(0.69–1.38)	0.9
AJCC N stage (7^th^)					
N0	751	Reference		–	–
N1	527	1(0.89–1.13)	0.966	–	–
N2	366	1.07(0.92–1.25)	0.359	–	–
AJCC M stage (7^th^)					
M0	1374	Reference		Reference	
M1	270	1.14(0.94–1.4)	0.191	0.91(0.73–1.14)	0.399
Regional nodes					
Positive	858	Reference		Reference	
Negative	786	0.45(0.27–0.75)	0.002	0.45(0.27–0.75)	0.002
Tumor deposits					
Positive	192	Reference		Reference	
Negative	1452	0.64(0.53–0.77)	<0.001	0.66(0.54–0.8)	<0.001
Bone metastasis					
Yes	2	Reference		–	–
No	1642	59963.62(0–Inf)	0.985	–	–
Liver metastasis					
Yes	197	Reference		–	–
No	1447	0.95(0.75–1.2)	0.659	–	–
Lung metastasis					
Yes	40	Reference		Reference	
No	1604	0.39(0.23–0.67)	0.001	0.45(0.26–0.8)	0.006
Perineural invasion					
Yes	248	Reference		Reference	
No	1396	0.79(0.66–0.93)	0.005	0.82(0.69–0.99)	0.035

*Two-sided P values <0.05; HR, hazard ratio; CI, confidence intervals; AJCC, American Joint Committee on Cancer; TNM, tumor-node-metastasis.

### Nomogram Validation

**Figure 3:**
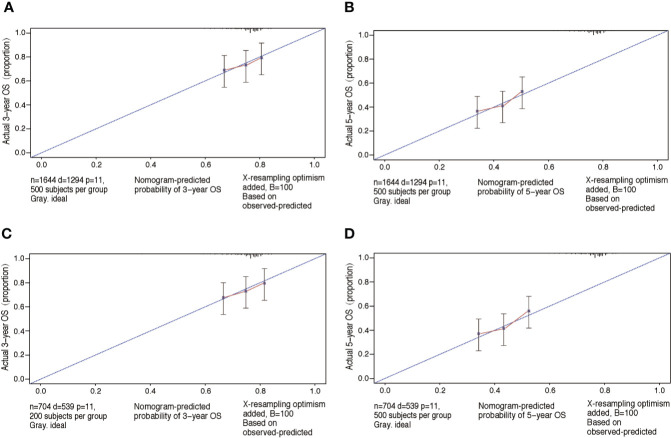
Calibration plots of OS associated nomograms in both training and validation sets. **(A, B)** Calibration plots of 3- and 5-year OS in training set; **(C, D)** calibration plots of 3- and 5-year OS in validation set. OS, overall survival.

**Figure 4:**
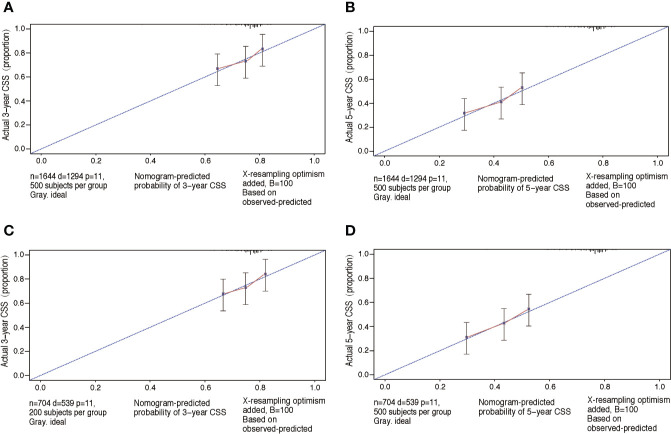
Calibration plots of CSS associated nomograms in both training and validation sets. **(A, B)** Calibration plots of 3- and 5-year CSS in training set; **(C, D)** calibration plots of 3- and 5-year CSS in validation set. CSS, cancer-specific survival.

**Figure 5:**
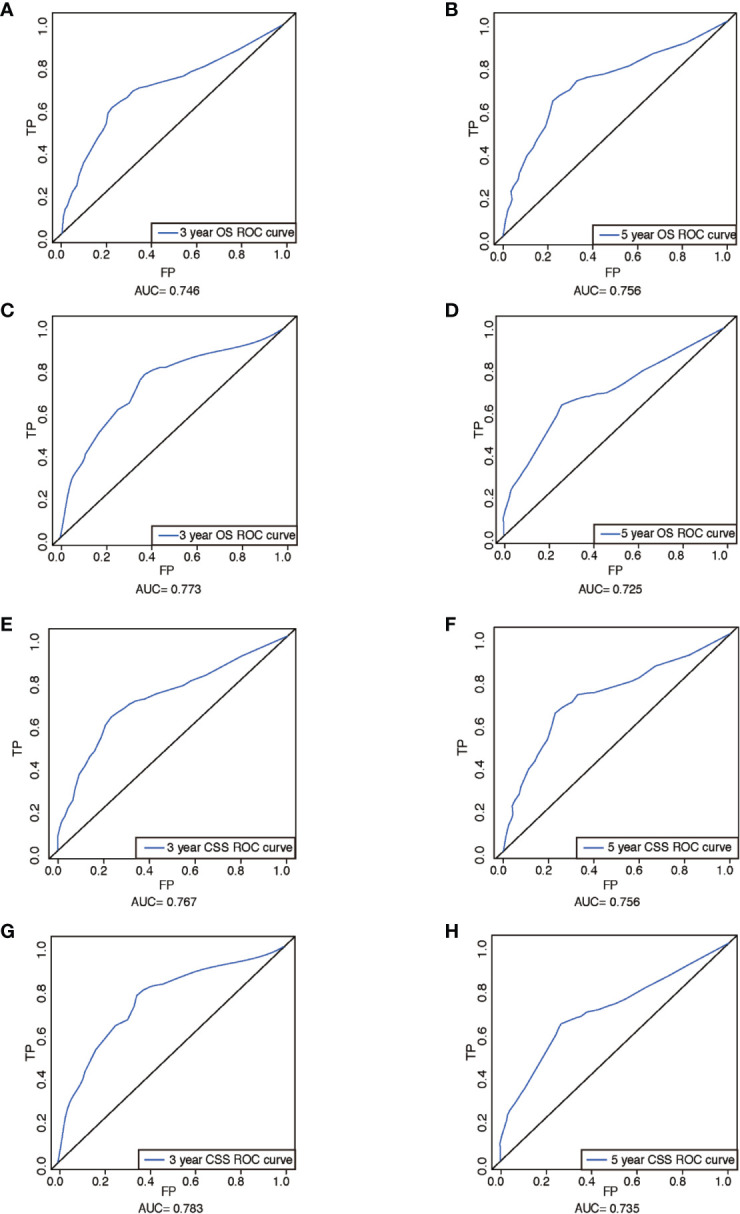
ROCs curve for the nomograms. **(A)** The ROC curve of nomogram with 3-year OS in training set; **(B)** the ROC curve of nomogram with 5-year OS in training set; **(C)** the ROC curve of nomogram with 3-year OS in validation set; **(D)** the ROC curve of nomogram with 5-year OS in validation set; **(E)** the ROC curve of nomogram with 3-year CSS in training set; **(F)** the ROC curve of nomogram with 5-year CSS in training set; **(G)** the ROC curve of nomogram with 3-year CSS in validation set; **(H)** the ROC curve of nomogram with 5-year CSS in validation set. ROC, receiver operating characteristic; OS, overall survival; CSS, cancer-specific survival; AUC, area under ROC curve; FP, false positive; TP, true positive.

**Figure 6:**
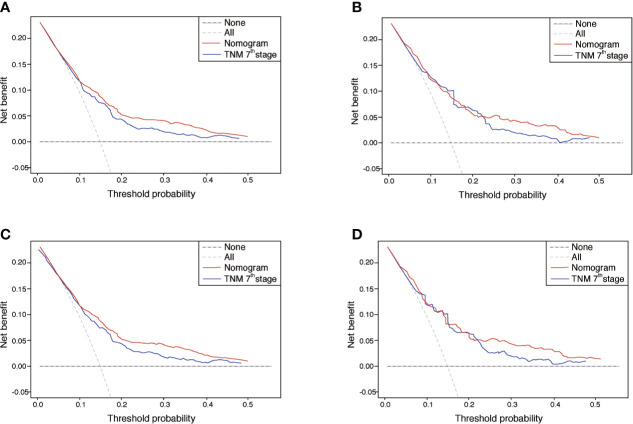
DCA of the nomograms for OS and CSS in both training and validation sets. **(A, B)** The DCA of nomogram in training set for both OS and CSS; **(C, D)** the DCA of nomogram in validation set for both OS and CSS. DCA, decision curve analysis; OS, overall survival; CSS, cancer-specific survival; TNM, tumor-node-metastasis.

The performance of nomograms was validated both internally and externally. When subjected to the internal validation, the nomogram exhibited predictive accuracy with C-index of 0.735 (95% CI: 0.708–0.762) for OS, and 0.765 (95% CI: 0.739–0.791) for CSS. In the external validation, the C-index for the OS nomogram was 0.736 (95% CI: 0.696–0.776), while for the CSS nomogram 0.76 (95% CI: 0.722–0.798). For the TNM staging system, the C-index to predict OS and CSS in the internal validation was 0.686 (95% CI: 0.662–0.711) and 0.712 (95% CI: 0.689–0.735), respectively. While in the external validation, the TNM staging system had a C-index of 0.68 (95% CI: 0.643–0.717) and 0.714 (95% CI: 0.695–0.733) to predict OS and CSS respectively, which indicated that the nomogram had better discriminatory ability than the traditional TNM staging system did. The calibration plots for the probability of 3-year and 5-year overall survival rate illustrated a fair agreement between the predicted probabilities and the observed proportions (**Figures 3**, **4**). The acceptable AUC values for the ROC curves were also noticed for prediction performance evaluation in training and validation sets, respectively (**Figure 5**). On decision curve analysis, the results indicated that nomograms showed a comparable clinical net benefit similar to 7th edition AJCC stage. The decision curve analysis was a novel evaluation method that assessed the clinical usefulness across different predictive models. In both the training and validation sets, OS nomogram displayed the better clinical net benefit almost over the entire range of threshold probabilities, while CSS nomogram was superior to TNM stage for both the training and validation sets when the threshold probability is greater than 26% (**Figure 6**).

## Discussion

The presented study developed OS and CSS prognostic nomograms for EOCA patients derived from the public database SEER. Through internal validation with bootstrap method and external validation, these nomogram models displayed favorable discrimination and calibration and comparable predictive performance to the TNM stage. The prognostic nomograms provided an alternative and complementary tool which would aid medical decision-making and follow-up scheduling as well as patient counseling. Our study extracted 2,348 eligible patients with EOCA from the SEER program which was a large population retrospective database. The patients were limited to those diagnosed between 2010 and 2015 considering the long-time span may have a certain impact on results. On the one hand, elderly patients with colon cancer are characterized by a significant decline in morbidity and potential mortality. This may lead to confounding biases in general prognostic indicators, especially when focusing on EOCA. On the other hand, the therapeutic strategies of colon cancer have been well standardized and improved over time, particularly the new breakthroughs of targeted therapy and immunotherapy (7).

We chose to focus on the nomogram of EOCA due to the following reasons. Young patients with colon cancer is a distinctive but common subset and the most frequent histological subtype being adenocarcinoma. The recent investigation found that young individuals under age 50 with colon cancer has shown a startling upward trend in need of greater emphasis and research (4). A previous study demonstrated that younger patients (≤ 40 years) have more aggressive more aggressive tumor biology with more advanced disease stages compared with older patients. However, younger patients often had a superior prognosis in overall survival and quality of life (8). Therefore, it was crucially important to identify key prognostic factors related to the survival time of patients with EOCA and establish an individualized and accurate survival prediction model for EOCA. Tumor survival prediction models are of great guiding significance for patient prognosis assessment, treatment regimens optimization, surgical patient screening, postoperative adjuvant treatment plan determination, identification of high-risk recurrence patients, follow-up frequency formulation and rational use of medical resources. Comparison with traditional TNM staging system, which only considers depth of tumor invasion, lymph node metastasis and distant metastasis, the nomogram prediction model with multiple factors were reported with major benefits (9). The nomogram transforms the complex regression equation into a visualized graph, which makes the results of the prediction model more readable and facilitates the evaluation of patients. It is precisely these inherent strengths that permit the application in medical research and clinical practice of nomograms.

A previous study by Zheng et al. has shown that the tumor deposits may be a significant indicators leading to the poor outcome for patients undergoing colon cancer resection surgery (10). Qi et al. have reported that tumor deposits was an independent unfavorable prognostic factor for DFS in N1-stage patients, associated with neural invasion and more common in young adults (11). Moreover, a recent study has indicated that the tumor deposits to be associated with negative prognostic effect, especially in stage IIIB colon cancer, with a 3.2-fold increased risk of disease recurrence (12). Also, female patients with colorectal cancer showed a slight but significantly better OS than men (13). Similarly, a meta-analysis by Yang et al. confirmed this finding when comparing nine studies (14). One possible explanation of better survival prognosis was that sex hormones may have a protective effect against colon cancer in young female patients (15). Additionally, numerous studies have validated the tumor size as a negative prognostic role. Dai et al. found that tumor size showed a considerable prediction value in T1 colon cancer, outperformed any other clinical prognostic factors (16). And, a recent study determined tumor size was positively correlated with T stage and negatively impacted survival (17). The findings from our analysis were in line with these previous reports.

However, we acknowledge that a number of variables, including age and race, did not show significant prognostic value in our study. This is reasonable since there were potentially valuable prognostic factors differences between EOCA patients and general colon cancer (CC) patients. In addition, the prognostic nomograms established in this study may not exhibit distinctly differences as compared to that of elderly CC patients. However, it was equally reasonable that regardless of the presence or absence of the difference between the EOCA nomogram and elderly CC nomogram, the prognostic performance of the nomograms in this study was not degraded.

Our study has the following advantages. First of all, the SEER database collects demographic characteristics, tumor characteristics, and survival data of populations in 17 regions across the United States, covering 28% of the US population, with data accuracy as high as 95% (18). This provides strong data support for the establishment of the nomogram, which is impossible to achieve in the general single-center study. Secondly, unlike previous nomograms built to predict the prognosis of patients with colon cancer, our models were more specifically targeted to assess the prognosis of colon adenocarcinoma patients under the age of 50 years. Finally, the calibration curves of the prognostic nomograms reached good concordance between the actual observation and the predicted probability, indicating that our models had good prediction ability.

Even so, there are some limitations in our study meanwhile. In this preliminary study, we obtained the data of EOCA patients from public transparency database and randomly assigned eligible cases into training or validation cohorts to evaluate the nomogram. Further validation in another independent population-based prospective cohort is still warranted before its routine clinical application. Additionally, some important clinical factors were not available in the SEER database including specific treatment information, smoking or alcohol drinking habits, etc. Moreover, the SEER database does not contain data on molecular markers, so it is difficult to evaluate the influence of these factors. These factors might have a potential impact on the effectiveness of the nomograms.

## Conclusions

The nomograms established in this study could effectively predict 3- and 5-year OS and CSS in EOCA patients, which assist clinicians evaluate prognosis more accurately and optimize treatment strategies for individual young patients.

## Data Availability Statement

Publicly available datasets were analyzed in this study. This data can be found here: Surveillance, Epidemiology, and End Results (SEER) database (https://seer.cancer.gov/).

## Author Contributions

HJ and YF contributed equally to this study. KG analyzed the data. HJ drafted the manuscript. SR contributed with a critical revision of the manuscript. All authors contributed to the article and approved the submitted version.

## Funding

National Natural Science Foundation of China (No.81573902); China Postdoctoral Science Foundation (No.2017M612040, No.2018T110610); Program for the Cultivation of Youth talents in China Association of Chinese Medicine (SR, No.QNRC2-C08, http://www.cacm.org.cn/); Zhejiang Provincial Program for the Cultivation of the Young and Middle-Aged Academic Leaders in Colleges and Universities (SR, No.2017-248, http://www.zjedu.gov.cn/); Zhejiang Provincial Project for the Key Discipline of Traditional Chinese Medicine (Yong Guo, No.2017-XK-A09, http://www.zjwjw.gov.cn/).

## Conflict of Interest

The authors declare that the research was conducted in the absence of any commercial or financial relationships that could be construed as a potential conflict of interest.

## References

[1] Siegel RL Miller KD Jemal A. Cancer statistics, 2020. CA: A Cancer J Clin (2020) 70(1):7–30. doi: 10.3322/caac.21590 31912902

[2] Kawai K Nozawa H Hata K Kiyomatsu T Tanaka T Nishikawa T. Nomogram Predicting Survival After Recurrence in Patients With Stage I to III Colon Cancer: A Nationwide Multicenter Study. Dis Colon Rectum (2018) 61(9):1053–62. doi: 10.1097/DCR.0000000000001167 30086054

[3] Mcmillan DC Wotherspoon HA Fearon KCH Sturgeon C Cooke TG Mcardle CS. A prospective study of tumor recurrence and the acute-phase response after apparently curative colorectal cancer surgery. Am J Surgery (1995) 170(4):319–22. doi: 10.1016/S0002-9610(99)80296-7 7573721

[4] Weinberg BA Marshall JL. Colon Cancer in Young Adults: Trends and Their Implications. Curr Oncol Rep (2019) 21(1):3. doi: 10.1007/s11912-019-0756-8 30659375

[5] Tricoli JV Boardman LA Patidar R Sindiri S Jang JS Walsh WD. A mutational comparison of adult and adolescent and young adult (AYA) colon cancer. Cancer (2018) 124(5):1070–82. doi: 10.1002/cncr.31136 PMC5821537 29194591

[6] Balachandran VP Gonen M Smith JJ Dematteo RP. Nomograms in oncology: more than meets the eye. Lancet Oncol (2015) 16(4):e173–80. doi: 10.1016/S1470-2045(14)71116-7 25846097 PMC4465353

[7] Banerjee A Pathak S Subramanium VD Dharanivasan G Murugesan R Verma RS. Strategies for targeted drug delivery in treatment of colon cancer: current trends and future perspectives. Drug Discovery Today (2017) 22(8):1224–32. doi: 10.1016/j.drudis.2017.05.006 28545838

[8] Rodriguez L Brennan K Karim S Nanji S Patel SV Booth CM. Disease Characteristics, Clinical Management, and Outcomes of Young Patients With Colon Cancer: A Population-based Study. Clin Colorectal Cancer (2018) 17(4):e651–61. doi: 10.1016/j.clcc.2018.06.007 30061036

[9] Shao N Xie C Shi Y Ye R Long J Shi H. Comparison of the 7th and 8th edition of American Joint Committee on Cancer (AJCC) staging systems for breast cancer patients: a Surveillance, Epidemiology and End Results (SEER) Analysis. Cancer Manage Res (2019) 11:1433–42. doi: 10.2147/CMAR.S185212 PMC6388984 30863154

[10] Zheng P Chen Q Li J Jin C Kang L Chen D. Prognostic Significance of Tumor Deposits in Patients With Stage III Colon Cancer: A Nomogram Study. J Surg Res (2020) 245:475–82. doi: 10.1016/j.jss.2019.07.099 31446189

[11] Qi QH Wang T Mao Y Hua D. Prognostic significance of tumor deposits in patients with stage III colon cancer. Chin J Oncol (2016) 38(10):784. doi: 10.3760/cma.j.issn.0253-3766.2016.10.015 27784466

[12] Landau MA Zhu B Akwuole FN Pai RK. Histopathological Predictors of Recurrence in Stage III Colon Cancer: Reappraisal of Tumor Deposits and Tumor Budding Using AJCC8 Criteria. Int J Surg Pathol (2019) 27(2):147–58. doi: 10.1177/1066896918787275 29992847

[13] Schmuck R Gerken M Teegen E Krebs I Klinkhammerschalke M Aigner F. Gender comparison of clinical, histopathological, therapeutic and outcome factors in 185,967 colon cancer patients. Langenbeck’s Arch Surg (2020) 405(1):71–80. doi: 10.1007/s00423-019-01850-6 32002628 PMC7036075

[14] Yang Y Wang G He J Ren S Wu F Zhang J. Gender differences in colorectal cancer survival: A Meta-analysis. Int J Cancer (2017) 141(10):1942–9. doi: 10.1002/ijc.30827 28599355

[15] Majek O Gondos A Jansen L Emrich K Holleczek B Katalinic A. Sex Differences in Colorectal Cancer Survival: Population-Based Analysis of 164,996 Colorectal Cancer Patients in Germany. PloS One (2013) 8(7):e68077. doi: 10.1371/journal.pone.0068077 23861851 PMC3702575

[16] Dai W Mo S Xiang W Han L Li Q Wang R. The Critical Role of Tumor Size in Predicting Prognosis for T1 Colon Cancer. Oncologist (2020) 25(3):244–51. doi: 10.1634/theoncologist.2019-0469 PMC7066694 32162825

[17] Saha S Shaik M Johnston G Saha S Berbiglia L Hicks M. Tumor size predicts long-term survival in colon cancer: an analysis of the National Cancer Data Base. Am J Surg (2015) 209(3):570–4. doi: 10.1016/j.amjsurg.2014.12.008 25601557

[18] Doll KM Rademaker AW Sosa JA. Practical Guide to Surgical Data Sets: Surveillance, Epidemiology, and End Results (SEER) Database. JAMA Surgery (2018) 153(6):588–9. doi: 10.1001/jamasurg.2018.0501 29617544

