# Corrigendum: Prognostic Nomograms for Predicting Overall Survival and Cancer-Specific Survival of Patients With Early Onset Colon Adenocarcinoma

**DOI:** 10.3389/fonc.2021.793652

**Published:** 2021-12-22

**Authors:** Huimin Jin, Yuqian Feng, Kaibo Guo, Shanming Ruan

**Affiliations:** ^1^ First Clinical Medical College of Zhejiang Chinese Medical University, Hangzhou, China; ^2^ Department of Medical Oncology, The First Affiliated Hospital of Zhejiang Chinese Medical University, Hangzhou, China

**Keywords:** nomogram, overall survival (OS), cancer-specific survival (CSS), early onset colon adenocarcinoma (EOCA), prognosis, SEER database

In the original article, there was a mistake in *“* **Figure 2** *| OS and CSS associated nomograms for EOCA patients”* as published. The contents of **Figure 2** and **Table 3** (including labels and data) correspond to each other, and there are no errors in **Table 3**. When we ran the code in the R software and exported **Figure 2**, some of the labels were not displayed completely and were manually added later. Due to our negligence, the gastric cancer stage “T1, T2, T3, T4a, T4b” was entered as “T1a, T1b, T2, T3, T4”. The corrected “**Figure 2** *| OS and CSS associated nomograms for EOCA patients”* appears below.

**Figure 2:**
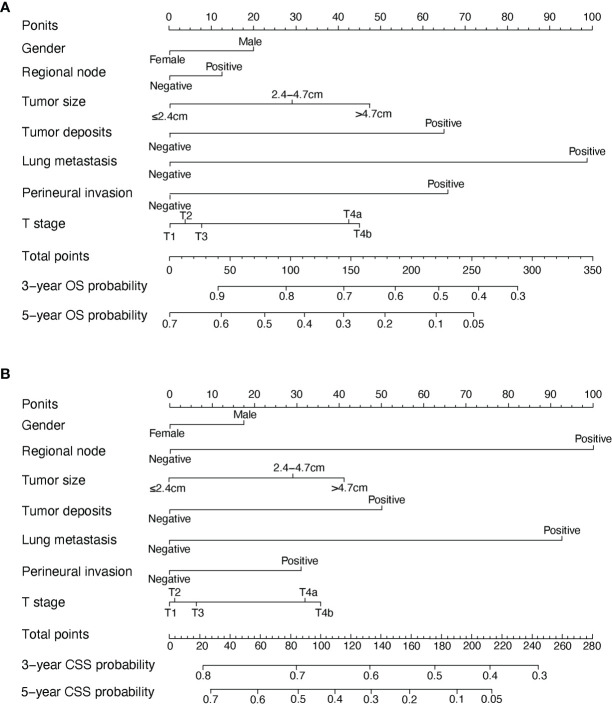
OS and CSS associated nomograms for EOCA patients. **(A)** OS nomograms for EOCA in 3- and 5-year; **(B)** CSS nomograms for EOCA in 3- and 5-year. OS, overall survival; CSS, cancer-specific survival; EOCA, early onset colon adenocarcinoma.

The authors apologize for this error and state that this does not change the scientific conclusions of the article in any way. The original article has been updated.

## Publisher’s Note

All claims expressed in this article are solely those of the authors and do not necessarily represent those of their affiliated organizations, or those of the publisher, the editors and the reviewers. Any product that may be evaluated in this article, or claim that may be made by its manufacturer, is not guaranteed or endorsed by the publisher.

